# The Host RNAs in Retroviral Particles

**DOI:** 10.3390/v8080235

**Published:** 2016-08-19

**Authors:** Alice Telesnitsky, Sandra L. Wolin

**Affiliations:** 1 Department of Microbiology and Immunology, University of Michigan, Ann Arbor, MI 48109, USA; 2 Department of Cell Biology, Yale School of Medicine, New Haven, CT 06536, USA; sandra.wolin@yale.edu

**Keywords:** non-coding RNA 1, HIV-1 2, retroviral assembly

## Abstract

As they assemble, retroviruses encapsidate both their genomic RNAs and several types of host RNA. Whereas limited amounts of messenger RNA (mRNA) are detectable within virion populations, the predominant classes of encapsidated host RNAs do not encode proteins, but instead include endogenous retroelements and several classes of non-coding RNA (ncRNA), some of which are packaged in significant molar excess to the viral genome. Surprisingly, although the most abundant host RNAs in retroviruses are also abundant in cells, unusual forms of these RNAs are packaged preferentially, suggesting that these RNAs are recruited early in their biogenesis: before associating with their cognate protein partners, and/or from transient or rare RNA populations. These RNAs’ packaging determinants differ from the viral genome’s, and several of the abundantly packaged host ncRNAs serve cells as the scaffolds of ribonucleoprotein particles. Because virion assembly is equally efficient whether or not genomic RNA is available, yet RNA appears critical to the structural integrity of retroviral particles, it seems possible that the selectively encapsidated host ncRNAs might play roles in assembly. Indeed, some host ncRNAs appear to act during replication, as some transfer RNA (tRNA) species may contribute to nuclear import of human immunodeficiency virus 1 (HIV-1) reverse transcription complexes, and other tRNA interactions with the viral Gag protein aid correct trafficking to plasma membrane assembly sites. However, despite high conservation of packaging for certain host RNAs, replication roles for most of these selectively encapsidated RNAs—if any—have remained elusive.

## 1. The RNA Content of Retroviruses

In the late 1960s, before RNA tumor viruses were known to reverse-transcribe and at a time when experiments characterizing RNA required radioactive cell labeling and cumbersome sedimentation gradients, it was already clear that RNA tumor viruses contained multiple RNA species: a high molecular weight class that was presumed to be the viral genome, and a heterogeneous pool of smaller RNAs with sedimentation coefficients from 4–10 S and unknown functions [1]. Now, almost 50 years later, we have extensive knowledge of the viral genome and recognize that some of the 4S RNAs are transfer RNAs (tRNAs) that prime reverse transcription. We also have a good understanding of the identities of most of the smaller encapsidated RNAs due to the application of RNA-sequencing (RNA-Seq) and other recent high throughput sequencing technologies [2,3]. However, the replication roles for most of the host RNAs in retroviruses remain enigmatic.

By mass, 30% or more of the RNA in retroviruses consists of host RNAs [4,5]. A small amount of messenger RNA (mRNA) is detectable in virion populations and the amount of packaged mRNA increases when the genomic RNA (gRNA) packaging signal is experimentally ablated [6,7]. However, although some mRNAs are more prominent than others among the host mRNAs in virions—with some rare mRNAs’ levels of enrichment rivaling that of gRNA for murine leukemia virus (MLV)—no mRNAs effectively compete with viral genomes for packaging, and the mRNA contribution to the total RNA in retroviral particles is ordinarily quite small [7,8,9].

In contrast, specific classes of small host non-coding RNAs—most of which are transcription products of RNA polymerase III (Pol III)—are abundant in retroviral particles. For example, one study that quantified relative levels of certain RNA species in virions and cells found that the host non-coding 7SL RNA is 250-fold more highly enriched in virions than actin mRNA [10]. Based on their total mass (about 1/3 or more of total virion RNA) and average length (very roughly 100 nucleotides (nt)), the host non-coding RNAs in retroviruses likely outnumber viral gRNAs by a factor of at least 50. Table 1 indicates the packaging numbers for certain host RNAs that have been reported in the literature. However, these numbers are fairly crude approximations, as measurements have been performed on virion populations, which may be heterogeneous, with copy numbers generally based on molar ratios relative to gRNA or 7SL as well as assumptions, such as that gRNA is packaged at a copy number of two per virion [8,11]. The first host RNAs packaged by retroviruses to be identified were tRNAs, some of which function in reverse transcription [12,13,14]. Other enriched ncRNAs are described below.

Subsets of endogenous retroelement RNAs expressed in virion producer cells also are abundantly packaged. These include intact endogenous retroviral transcripts like the murine VL30 elements that are packaged by MLV, and fragments of non-long terminal repeats (LTR) retroelements such as the transcripts of divergent and truncated Long INterspersed Elements (LINEs) packaged by human immunodeficiency virus 1 (HIV-1) [3,9,21]. Recombination between endogenous and exogenous retroviruses contributes to some animal retrovirus pathogenesis, and the possibility that retroelements might be transduced in place of retroviral vectors or contribute to the generation of replication competent retroviruses has been expressed as a concern of retroviral gene transfer [22]. However, not all endogenous retroelements are packaged by all retroviruses. For example, although HIV-1 infection appears to enhance expression of human endogenous retrovirus K (HERV-K) elements, human endogenous retroviral RNAs do not appear to be packaged by HIV-1 [23,24].

What about microRNAs (miRNAs)? Deep sequencing studies of the shorter RNAs in HIV-1 particles report that some miRNAs are selectively packaged [25]. However, unlike that of other RNA viruses, retroviral replication does not entail generation of extensive double-stranded RNA, and for at least most retroviruses, the evidence that miRNAs may be encoded in their genomes remains controversial [26,27,28].

### 1.1. Host ncRNA in Retroviruses: tRNAs

The roles of specific encapsidated tRNAs in reverse transcription were first described in the 1970s [12,13,14], and primer tRNAs are the only encapsidated host RNAs with undisputed viral replication functions. Primer tRNAs are so named because their 3′ ends, annealed to 18 nucleotides of complementarity near the 5′ ends of viral genomic RNA, serve to initiate reverse transcription upon uncoating of retroviral particles early in infection [29]. Retroviruses differ in which tRNA they use to prime reverse transcription. For example, HIV-1 uses lysine tRNAs (specifically the Lys3 isoacceptor), MLV uses tRNA-Pro, and Rous sarcoma virus (RSV) uses a tryptophan tRNA as primer [30,31,32]. The total number of tRNAs per virion has been assigned values ranging up to about 50 per virion, with, for example, 8–12 copies of the Lys3 primer tRNA per virion reported for HIV-1 [4,5,16]. The tRNAs that prime reverse transcription are encapsidated in infecting virions and not recruited from infected cells. Initial interactions between tRNA and gRNA are weak, and primer annealing is not complete until after virion maturation and viral nucleocapsid (NC) protein-chaperoned remodeling of gRNA’s primer binding site and the primer tRNA [33].

The spectra of packaged tRNAs differ from those observed in cells, with both the tRNA used to prime reverse transcription and certain non-primer tRNAs enriched over other tRNAs about 10-fold relative to their intracellular levels for HIV-1, and differing levels of enrichment among retroviral genera [11,34]. For example, non-primer lysine tRNA isoacceptors are enriched in HIV-1 in addition to the Lys3 primer tRNA [35,17], and whereas these non-primers do not function in reverse transcription, they may contribute to the nuclear import of HIV-1 replication intermediates [36]. tRNA synthetases contribute to tRNA recruitment for at least some types of retroviruses. For example, interactions between HIV-1’s primer tRNA and its cognate tRNA synthetase are necessary for primer recruitment, since tRNA mutants defective in synthetase interactions are not packaged [37]. A comparison of HIV-1 virions to Gag-only virus-like particles (VLPs) suggests that both the amount and the characteristic spectrum of tRNAs in HIV-1 may rely on GagPol [17]. A current model for tRNA packaging suggests that reverse transcriptase sequences in the context of GagPol bind tRNAs non-specifically, and that these interactions and additional interactions between Gag and tRNA synthetases in the cytoplasm lead to observed virion tRNA populations [38,39]. However, this model is almost certainly over-simplistic, as several examples of specific non-primer tRNAs that are rare in cells but enriched in virions have been reported [17].

### 1.2. 7SL RNA

Early analysis of RSV suggested that about a quarter of its total host RNA content consisted of a single 7S RNA species in 5- to 10-fold molar excess to the 70S presumptive genome [18]. The observation that 7S RNA was an abundant component of avian and murine retroviruses, and subsequent observations that the same RNA was a component of normal cells’ polyribosomes, represented the initial discovery of what is now known as 7SL or signal recognition particle (SRP) RNA [40,41,42,43]. On a molar basis, 7SL is by far the most abundant non-tRNA species in all retroviruses whose RNA content has been determined, with encapsidated 7SL copy numbers exceeding those of genomic RNA by a factor of about six by most measures [2,44], although one study reported 7SL and genomic RNA are present in equimolar amounts [7]. 7SL RNA has been studied fairly extensively in the case of HIV-1, where it is encapsidated in proportion to Gag and observed in virus-like particles in indistinguishable molar ratios whether Gag is full length or present only as a minimal assembly-competent form comprised of the C-terminal domain of capsid protein (CA) plus the adjacent spacer peptide, SP1 [10,45] (although again, there is some dispute over these findings in the literature [46]).

7SL is the RNA component of signal recognition particles, the ribonucleoprotein (RNP) that ferries nascent signal peptides to the endoplasmic reticulum and mediates co-translational export of secretory proteins [47]. Although SRP RNA was initially believed to serve simply as a scaffold for the six SRP proteins that bind it, it is now clear that 7SL is a dynamic component of SRP [48]. The RNA itself performs functions, such as accelerating the interaction of signal peptide-bound SRP with its receptor and stimulating guanosine triphosphate hydrolase (GTPase) activities, which are typically performed by protein catalysts [49]. The highly dynamic character and changing properties of 7SL RNA throughout the process of co-translational protein targeting, such as the molecular gymnastics performed to position and relocate protein partners as they perform their functions, lend credibility to the speculation that this RNA could be commandeered to assist assembly processes during viral replication. However, to date, no roles for 7SL in retrovirus replication have been uncovered.

Intriguingly, the Alu family of repetitive sequence elements that pepper our genomes are comprised of portions of the 7SL gene [50]. Alu elements are non-autonomous retroelements that are mobilized by the reverse transcriptase of LINEs [51]. The fact that 7SL RNA is recruited by exogenous retroviruses while its Alu derivatives are mobilized by LINEs suggests that 7SL recruitment could reflect a highly conserved interaction. This link is further supported by observations that the ancestral RNAs of several additional classes of short interspersed elements (for example, tRNAs, 5S ribosomal RNA (rRNA), and U6) are also prominently packaged by retroviruses [52,53].

### 1.3. Other Packaged Non-Coding RNAs

Besides 7SL and tRNAs, packaged host RNAs that are present in at least one copy per virion for one retrovirus or another include the 60 kDa Ro autoantigen-associated RNAs Y1 and Y3, which are each present at about four copies per MLV virion [20], the spliceosomal RNA U6, which is present at about one copy per MLV or RSV virion [8,19], 5S rRNA in MLV [8] and vault RNA, which is present at about one copy per MLV [9]. Like tRNAs and 7SL RNA, one property that each of these enriched RNAs shares is that it is a RNA Pol III transcript. Several other host RNAs appear enriched within virions, in that they are present at an order of magnitude or more higher concentrations in virions than in cells when normalized to actin mRNA. These later RNAs, which are present at less than one copy per virion, include several spliceosomal RNAs other than U6 as well as several other small noncoding RNAs [3,8,9].

## 2. Host RNAs Are Not Packaged at Random

The trafficking and molecular associations of replication intermediates that form between the time of provirus transcription and the appearance of assembly intermediates at the plasma membrane remain poorly understood and are areas of active investigation. Multimerization of a small number of Gag molecules on genomic RNA is reported to precede gRNA arrival at the plasma membrane [54,55]. However, although it has been known for over a decade that HIV-1 RNA dimerization happens after nuclear egress, questions as fundamental as whether gRNA dimerization takes place within the cytoplasm or at the plasma membrane remain unresolved [56,57,58]. Where ncRNAs join the assembling subviral nucleoprotein complex is understood even less well than the interactions of gRNAs with each other.

In a simplified view of HIV-1 and murine and avian retroviruses’ assembly, virions form when coalesced Gag molecules on the plasma membrane pinch off to form membrane-bound particles. Describing retroviral assembly in this way suggests the possibility that packaged host RNAs might represent a random sampling of the cytoplasm. However, it is clear from a comparison of virion and cytoplasmic RNA content that retroviruses are not merely packets of cytoplasmic contents [8]. For example, while all tRNA^Lys3^ detectable in cells is aminoacylated, this host RNA—as found within HIV-1 virions—is not aminoacylated [16].

Another striking example of the non-randomness of host RNA packaging comes from studies of host Y RNA packaging by MLV [20]. Y RNAs, together with a 60-kDa protein called Ro, comprise the highly abundant cytoplasmic Ro RNP. This host cell RNP, which was discovered as a frequent target of autoimmune responses, may function in noncoding RNA quality control [59]. Y RNAs are unstable without Ro, and Y RNAs are detected in Ro protein knockout cells at only a few percent of their normal levels in isogenic Ro^+/+^ cells, even when Y RNA expression is not directly affected [60,61]. When Ro knockout cells were MLV-infected, viral replication was unperturbed. Remarkably however, the amount of Y RNA encapsidated in virions produced from Ro knock-out cells remained at the same high levels as was observed for Ro^+/+^ cells, despite the virtual absence of Y RNA within knockout producer cells [20]. This suggests that Y RNAs are recruited for MLV packaging at a very early stage of their biogenesis: after Y RNA transcription, but before those Y RNAs that are not associated with Ro protein can be degraded.

## 3. Determinants of ncRNA Packaging

The determinants of ncRNA packaging differ from those of gRNA. For example, MLV particles formed by viral NC protein mutants with alterations in residues responsible for specific recruitment of gRNA retain wild-type levels of 7SL [10,62]. Additionally, ncRNA packaging levels are largely if not entirely independent of gRNA packaging, indicating that their packaging does not require ncRNAs to base-pair or otherwise interact with gRNA for their initial recruitment [8,10,63]. However, despite the absence of extensive complementarity between viral genomes and any of the abundantly packaged host RNAs other than primer tRNAs, early reports described heat-disruptable interactions between purified viral and co-packaged host RNAs, and it is possible base-pairing interactions form during virion maturation [12,41,64].

The Y RNA findings from Ro knockout cells described above suggested that Ro was not necessary for Y RNA encapsidation, and indeed Ro protein is not detectable in MLV particles [20]. The inference that packaged host RNAs are encapsidated shortly after their synthesis, before their assembly into mature RNPs, is strengthened by observations with 7SL. SRP54 protein recruitment occurs in the cytoplasm and is the final step in SRP assembly, while most earlier steps in SRP biogenesis take place in nucleoli [65,66,67]. Whereas nearly all intracellular 7SL RNA exists in mature SRP particles, HIV particles lack detectable SRP54 protein [10,68]. The cognate proteins of other prominently encapsidated RNP RNAs, such as telomerase associated protein 1 (TEP1)—the major protein component of vault RNPs—and SRP19—the SRP subunit whose binding to 7SL allows SRP54 association—also are not detected in virions, demonstrating that the encapsidation of ncRNAs outside their major mature RNPs is a generalized feature [3,9,20].

Studies of 7SL packaging support a model in which 7SL interacts with multiple regions in Gag. Eukaryotic 7SL RNA is comprised of two folded domains called the Alu and S domains, separated by a linker region [69]. Because 7SL RNA is packaged by minimal assembly-competent HIV-1 VLPs, the viral determinants of its incorporation into HIV-1 particles map to the carboxy-terminal domain of CA plus spacer peptide SP1 [10]. However, these portions of Gag may or may not interact with 7SL directly. In this regard, it may be noteworthy that SRP14 is one of the top 50 host proteins identified as Gag interactors [70]. Additionally, in the absence of the NC domain of Gag, 7SL is detectable in VLPs as a ~100 bases S domain fragment called 7SLrem, thus suggesting that 7SL is ordinarily protected from a nuclease present in VLPs by sequences within the NC domain [45]. NC interactions with 7SL RNA have also been described in crosslinking studies [71].

The *cis*-acting determinants of 7SL packaging by HIV-1 have been addressed by examining the packaging of exogenously expressed 7SL RNA mutants [72]. These analyses showed that a 114 nt RNA comprising the Alu domain was packaged as well as full length 7SL, but that smaller Alu derivatives—while well-expressed—were not packaged. Surprisingly, a non-overlapping second portion of 7SL—containing the S domain—also was packaged efficiently. However, whereas an overexpressed Alu domain competed with 7SL for packaging, encapsidation of overexpressed S domain occurred additively [72].

A possible significance of even the substoichiometric ncRNAs found in virions is that their recruitment may be mementos of the trafficking pathways taken by viral subassemblies, and that their study may illuminate early pathways in retroviral assembly. Observations that the spectra of packaged RNAs change under conditions wherein host ncRNA traffic or RNP assembly pathways are altered may indicate that the RNAs are recruited into assembling viral RNPs in competition with their normal biogenesis and decay pathways [3,9]. As further described below, retrovirus ncRNA populations are enriched in unusual, ordinarily transient, forms of host RNAs that suggest an intersection between retroviral assembly and host ncRNA biogenesis and surveillance pathways [3,9].

In at least some reports, the spectra of host ncRNAs identifiable in extracellular vesicles released by uninfected cells are similar to those reported for retroviruses [73], leaving open the possibility that some host RNAs reportedly packaged by retroviruses are instead in extracellular vesicles and not virions. However, the above findings that certain mutations to 7SL render RNA non-packageable yet highly expressed provide confirmation that ncRNAs are bona-fide virion components. Additional evidence that these RNAs reside within virions includes the co-migration of viral genomes and host RNAs on density gradients, orders of magnitude higher levels of host RNA in virus fractions than similar fractions of media from uninfected cells, and the occasional generation of a host RNA reverse transcription product [1,3,8,74,75].

## 4. Host RNP Biosynthesis and Retroviral Assembly

At least some host ncRNAs are demonstrably packaged as nascent RNAs [3,9]. A high throughput sequencing analysis of the RNA content of gradient purified MLV revealed that precursor forms of specific tRNAs, and of small nuclear and small nucleolar RNAs, were much more abundant relative to their mature forms in the encapsidated virion RNA population than within the cells that produced them [9]. In fact, the level of enrichment of at least some of these RNAs is greater than 7SL’s. A similar spectrum of pre-tRNAs and small nuclear RNAs (snRNAs) was observed in HIV-1 [3]. In cells, these precursor RNAs are generated and subsequently matured in the nucleus, which makes their inclusion in viruses that bud from the plasma membrane all the more surprising [76].

However, despite molecular signatures suggesting they are nuclear products, at least some of these RNA processing intermediates appear to be recruited for packaging from the cytoplasm. Specifically, by blocking nuclear re-entry of snRNA U2 precursors, which undergo nuclear export and subsequent re-import as part of their normal biogenesis, packaging of these very rare ncRNA species increased. Similarly, depletion of receptors required for nuclear export of certain ncRNAs decreased encapsidation of the corresponding ncRNAs [3,9]. Additional findings that A- and U-tailed forms of the ncRNAs accumulated upon depletion of processing exoribonucleases suggest nascent retroviral RNPs intersect with a previously unrecognized host ncRNA surveillance pathway during preassembly stages of replication.

## 5. Possible Roles of the Host RNAs Packaged by Retroviruses

As described above, host tRNAs function in the initiation of reverse transcription by serving as primers. Although no other essential replication roles have been demonstrated for the encapsidated host RNAs, the following discusses occasional and postulated roles.

### 5.1. Non-Priming Roles in Reverse Transcription

A key step in retroviral replication is the generation of a DNA copy of encapsidated viral RNA. Ordinarily, viral gRNA serves as the reverse transcription template, but exceptions have been observed. For example, reverse transcription products of encapsidated VL30 retroelements are readily detectable among MLV endogenous reaction products [77]. RNAs that lack packaging signals can at least occasionally become co-packaged with simple retrovirus gRNAs, because they have been observed to serve as recombination substrates [78,79]. Furthermore, a RSV nucleocapsid mutant defective in gRNA packaging was observed to transduce a selectable marker gene by generating a processed pseudogene from an encapsidated non-viral RNA [80].

Reverse transcription products of 7SL have been observed in endogenous reactions and patched into an experimental recombinant [74,81]. Although their integration site signatures indicate at least many are generated by LINE element replication machinery, it has been suggested that retroviruses may be capable of using packaged snRNAs to generate pseudogenes [19,74,81].

Sometimes portions of encapsidated host RNAs contribute to reverse transcription products via recombination. Retroviral recombination is exceptionally frequent, and results from template switching between RNA templates during reverse transcription [82]. A classic example of host sequence recombination is the insertion of host oncogenes into acute transforming retroviruses: a process that is believed to involve non-homologous recombination between viral RNA and host sequences that become co-encapsidated due to proviral polyadenylation signal readthrough [83]. In other cases, patched-in sequences can be genetically unlinked to the parental proviruses’ integration site, likely reflecting the co-packaging of viral genomes and host RNAs [84]. Retroviral recombination between unrelated sequences often requires a tertiary template, and can result in “patch repair” using host sequences with fortuitous microhomology to splint across non-homologous recombination junctions [85,86,87]. In some cases, the resulting inserted sequences have provided functions or served as the starting points for further viral evolution [82]. In a remarkable example of this, recombination between HIV-1’s reverse transcriptase gene and slightly different portions of the identical human host sequences—in two separate infected patients located continents apart—resulted in sequence insertions that conferred RT drug resistance [88,89].

### 5.2. Host RNAs’ Possible Roles in Trafficking and Assembly

There are marked differences in the sensitivity to ribonuclease (RNase) of immature and mature virions, and among retroviruses. For example, uncoated immature MLV particles are sensitive to RNase treatment but those of HIV-1 are not [90,91,92]. Nonetheless, the behavior of RNA-binding defective viral mutants and observations such as the capability of RNA to drive assembly of virus-like particles in vitro has led to a general consensus that RNA contributes to assembly and/or is critical to the structural integrity of retroviruses [6,93]. Based on observations in vitro where addition of essentially any single-stranded nucleic acid can promote Gag assembly, and the fact that expression of Gag alone in mammalian cells is sufficient for the generation of virus-like particles, it has been suggested that non-specific interactions between Gag and RNA drive retroviral assembly [54,94,95,96]. Here, “non-specific” refers to electrostatic interactions between multiple basic residues in the relatively unstructured regions of NC and nucleic acids, as opposed to the specific interactions that mediate gRNA recruitment and can be largely ablated by specific mutations to NC zinc finger motifs. Although many different molecules can provide these non-specific assembly functions under reconstituted reaction conditions, it seems likely that the RNAs encountered during assembly in cells are more defined, and that retroviruses may have evolved to use specific RNAs.

Retroviral particles are released from cells with indistinguishable kinetics whether or not gRNA is available, but retroviral assembly requires RNA [90,97]. Thus, it seems possible that host RNAs may function in assembly, or contribute to uncoating during early replication steps. Because 7SL RNAs are the most highly conserved host RNAs in retroviruses other than tRNAs, they seem like obvious candidates [2]. However, despite its conservation in retroviruses and retroelements, the case for 7SL playing an irreplaceable replication role is all but ruled out by the observation that artificial depletion of 7SL does not reduce virion infectivity. Specifically, neither overexpression of 7SL fragments nor overexpression of SRP19 reduces infectivity appreciably, although both treatments reduce virion 7SL content [72,98,99,100]. However, other reports seeking to define elements required for virion assembly have reported apparent functional redundancy in factors required for particle assembly [46]. Such lines of thinking leave open the possibility that 7SL or other host RNAs may ordinarily contribute to assembly but that viruses can compensate for their absence.

Gag undergoes a complex series of RNA binding events during assembly [54,93]. With no host RNA specifically implicated in these events, perhaps the simplest hypothesis is that viral gRNA itself is both specifically recruited and then—because it exists in high local concentration—serves non-specific assembly roles, and that host RNAs can readily provide these later functions when gRNAs are absent. This model may be consistent with recent cross-linking studies probing Gag-gRNA interactions during HIV-1 assembly, which show that interactions are largely focused on the packaging element and a couple other regions of the genome prior to assembly, but that NC domain binds far more broadly to many gRNA sites after maturation [71].

Despite lack of evidence for any encapsidated host RNAs functions, recent findings demonstrate that specific host ncRNA do play important replication roles prior to HIV-1 virion release [71]. Crosslinking-immunoprecipitation (CLIP) and sequencing of target RNAs throughout virion biogenesis revealed that the predominant RNA species bound to Gag within cells were tRNAs and 7SL. Surprisingly, while 7SL and mRNA reads mapped to NC, the tRNAs were bound to matrix (MA).

The finding that most intracellular interactions between Gag and RNA map to MA was surprising. For retroviruses like HIV-1 and simple murine and avian retroviruses, Gag selectively multimerizes at the plasma membrane and not on intracellular membranes such as those of endosomes. This targeting maps to the highly basic region of the matrix domain of Gag and requires interactions with specific membrane phospholipids [101,102,103,104]. It has been proposed that these basic regions of MA may interact with viral or cellular RNAs before reaching the plasma membrane, and that this may limit associate with intracellular membranes during Gag trafficking. The new CLIP studies [71] support the model that MA can bind RNA or the plasma membrane but not both simultaneously, and that reversible host tRNA binding to MA’s basic patch plays a critical assembly function in that it helps regulate Gag localization [105,106].

The assembly steps that precede membrane binding are among the least-well understood stages of retroviral replication, and likely not only include the stages where host RNAs are recruited, but may also include steps that are affected by encapsidated host RNAs. With the absence of visible intracellular Gag foci, arguably most evidence suggests Gag multimerization occurs primarily at the plasma membrane [107], with gRNA trafficking to the membrane in complex with a small number (perhaps 12) of Gag molecules, where the gRNA complex is joined by the bulk of Gag that will comprise the budding virions [97]. However, other work has provided evidence supporting a model in which HIV-1 assembly and packaging occurs in a subclass of host RNA granules defined by their possession of RNA DEAD-box helicase 6 (DDX6), which are not processing bodies (P-bodies) or stress granules [108,109]. Further understanding of these RNA-granule like assembly intermediates may aid understanding of how host RNAs end up in virions.

Some host tRNA species have also been implicated in facilitating an early step in HIV-1 replication. Specifically, in one study that sought to identify host factors that promoted nuclear entry of HIV-1 preintegrative reverse transcription complexes, (possibly aberrant) tRNAs were shown to be the active component of a HeLa cytosolic extract fraction [36]. Interestingly, although most MA is known to dissociate from reverse transcription complexes shortly after initial infection [110], about half of the most abundant tRNAs found associated with MA in the above CLIP studies were also among the subset of tRNAs found to promote HIV-1 reverse transcription complex nuclear entry (compare Supplementary Figure S6 in [71] to Table 1 in [36]).

The endogenous retroelement RNAs that retroviruses package are undoubtedly not necessary for virus replication, since they are encapsidated at less than one copy per virion when produced by cells that express them, and infectious virions can be produced by cells that do not express these RNAs. However, some evidence suggests retroelement RNA sequences may be able to substitute for required assembly functions under certain circumstances. Specifically, in one study aimed at identifying host factors required for retroviral assembly that involved selecting mutagenized cell pools for clones resistant to murine leukemia virus infection, subsequent selections identified suppressor genes capable of restoring virus susceptibility [111,112]. Surprisingly, both of the highly potent suppressor genes identified appeared to consist of non-coding RNA fragments, one of which was a portion of an endogenous VL30 retroelement [112]. Both the mechanisms of MLV resistance and how the identified RNAs overcome this suppression remain to be determined.

### 5.3. Scaffolds for RNA Binding Factor Recruitment

The discovery that members of the retroviral restriction factor apolipoprotein B mRNA editing enzyme, catalytic polypeptide 3 (APOBEC3) family of cytidine deaminases—most notably APOBEC3G (A3G)—are RNA binding proteins ignited interest in deciphering which kind of RNA these intrinsic antiviral factors use to gain access to the virion. There is general agreement that the incorporation of A3G requires RNA binding and that this RNA is ordinarily recruited via interaction with Gag’s NC domain [75,113,114]. However, the identity of the RNA responsible has been controversial, with gRNA and 7SL alternately being reported to be necessary for A3G recruitment, while others have suggested either or both could be depleted and A3G still packaged [68,98,99,100,113]. More recent studies have suggested that A3G is a fairly non-specific RNA binding protein that can recognize relatively short unstructured motifs [115], and that any of a number of RNAs are competent to recruit A3G, with NC itself not required [100].

### 5.4. Innate Immune Sensing

Another speculative role of encapsidated host RNAs is in host sensing of retroviral infections. If so, it is possible that the conserved packaging of host Pol III products does not reflect a selective advantage to the virus but instead is an evolved defense by the host. The 5’ ends of Pol III transcripts are not 7-methylguanylate capped, but rather triphosphorylated, and thus should be recognized by cytosolic RNA sensors such as retinoic acid inducible gene 1 (RIG-I), which activate signaling cascades that lead to the expression of cytokines such as interferon (IFN). IFN-α can suppress HIV replication in patients, and IFN-stimulated genes are upregulated in infection, including several antiviral restriction factors that limit HIV replication [116].

Recent screens for the mediators of HIV-1 molecular sensing have principally implicated sensors of reverse transcription products (DNA) [117,118,119]. There is nonetheless at least some evidence that retroviral RNA is sensed by the host. It has long been noted that retroviral gRNAs are nicked when extracted from virions, leading to speculation that the encapsidation of a dimeric genome may have evolved in part as a defense against a host antiviral nuclease [82,120]. In the absence of NC protein, encapsidated host ncRNAs are subject to partial degradation as well [45]. Whereas the fates and associations of host ncRNA upon virion uncoating are unknown, both HIV-1 and MLV have been reported to activate Toll-like receptor 7 (TLR7) [121,122], and as in vitro transcripts, some of the ncRNAs packaged by retroviruses are known to be potent TLR7 ligands [123]. Provocatively, certain HIV-1-derived Gag VLPs reported to generate enhanced immune responses in mice were found to contain enhanced levels of Y1 and other ncRNAs [124,125].

### 5.5. Possible Practical Implications of Host RNA Packaging

With little evidence of relevance to retroviral biology, is there any significance to the packaging of host ncRNAs? In the course of defining *cis*-acting determinants of 7SL packaging, it was observed that overexpressed S domain RNAs were packaged in addition to, rather than in competition with, endogenous 7SL [72]. When varying ratios of a plasmid expressing the S domain were co-expressed with a plasmid expressing HIV-1 proteins, S RNA packaging levels changed accordingly, and S RNA copy numbers of 100 per virion were readily achievable [72]. This suggests that retroviruses’ ability to encapsidate large numbers of small RNAs could be adapted to promote delivery of specific RNAs to targets of infection.

## 6. Summary

When retroviruses assemble, the RNA they incorporate is 30%–50% host derived by mass, likely representing roughly 100 host RNA molecules per virion. Some of these are tRNAs recruited directly or as a consequence of the machinery necessary for primer tRNA packaging. Others are endogenous retroelement RNAs that likely share packaging mechanisms with the virus genome. The most abundantly packaged host ncRNAs are unusual forms of RNA Pol III transcripts: these lack their normal protein binding partners and often are processing intermediates or show other signatures of recruitment from early phases of their biosynthesis. Despite their abundance and the high conservation of packaging of some host ncRNAs in a uniform stoichiometry relative to Gag, the replication roles these RNAs remain elusive.

## Figures and Tables

**Table 1:** Some of the RNAs in retroviral particles.

RNA	Approximate Copy Number/Virion	Virus That Harbors	References
gRNA (viral)	2	MLV, HIV, RSV, etc.	[15]
Host mRNA	0.05 ^1^	HIV-1 (e.g., actin mRNA)^1^	[10]
Primer tRNA	8	HIV-1	[16]
Other lysyl tRNAs	12	HIV-1	[16]
All tRNAs	50	HIV-1, RSV, etc.	[1,17]
7SL RNA	12	MLV, HIV, RSV, etc.	[8,10,18]
U6 snRNA	1	MLV, RSV	[8,19]
Y RNAs	4	MLV	[8,20]
Vault RNA	1	MLV	[9]

^1^ Based on [10] and assumption that actin mRNA is 10-fold more abundant in infected cells than HIV-1 RNA. MLV: murine leukemia virus; HIV: human immunodeficiency virus; RSV: Rous sarcoma virus; mRNA: messenger RNA; tRNA: transfer RNA; snRNA: small nuclear RNA
